# Comorbid Insomnia and Sleep Apnea Mechanistic Convergence, Phenotypic Heterogeneity, and Clinical Implications

**DOI:** 10.1007/s40675-026-00384-w

**Published:** 2026-06-18

**Authors:** Syed Nahidi, Senyo Agidi, Ali A. El-Solh

**Affiliations:** 1 Division of Sleep Medicine, The Veterans Affairs Western New York Healthcare System, New York, USA; 2 Department of Medicine, Jacobs School of Medicine and Biomedical Sciences, New York, USA; 3 Department of Epidemiology and Environmental Health, School of Public Health and Health Professions, New York, USA; 4 Department of Anesthesia, Jacobs School of Medicine and Biomedical Sciences, New York, USA; 5 Medical Research, VA Western New York Healthcare System, Bldg. 20 (151) VISN02, 3495 Bailey Avenue, NY 14215 – 1199 Buffalo, USA

**Keywords:** COMISA, Insomnia phenotypes, PAP, CBT-I, Dual orexin receptor antagonists

## Abstract

**Purpose of Review:**

Comorbid insomnia and obstructive sleep apnea (COMISA) is increasingly viewed as more than two coexisting sleep disorders. This review asks whether COMISA reflects a distinct phenotype driven by mechanistic convergence, how heterogeneous it is across patients, and what that means for diagnosis and treatment.

**Recent Findings:**

Recent studies show COMISA is common, linked to greater sleep fragmentation, and higher cardiometabolic and psychiatric burden than either disorder alone. Mechanistic work supports bidirectional interaction between ventilatory instability and chronic hyperarousal, with autonomic and EEG findings suggesting persistent sympathetic activation and cortical hyperactivation. Treatment studies indicate that PAP and CBT-I each help, but single-modality treatment often leaves residual symptoms; combined, phenotype-informed care appears more effective.

**Summary:**

The evidence supports COMISA as a clinically important, heterogeneous convergence phenotype rather than a simple comorbidity. Future research should prioritize longitudinal outcomes, biomarker-based phenotyping, and endotype-stratified trials. This shift could move COMISA care toward more precise, mechanism-guided management.

## Introduction

Comorbid insomnia and obstructive sleep apnea (COMISA) represent the coexistence of two of the most prevalent sleep disorders, yet accumulating evidence suggests that this overlap reflects more than incidental comorbidity. Insomnia symptoms are reported in approximately 40–60% of patients with obstructive sleep apnea (OSA), and 30–40% of individuals presenting with chronic insomnia meet diagnostic criteria for OSA when systematically evaluated [1, 2]. Despite this substantial overlap, insomnia and OSA are often assessed and managed within separate diagnostic pathways, contributing to underrecognition of their interaction and incomplete therapeutic response. Contemporary literature increasingly supports the view that COMISA constitutes a distinct and clinically consequential phenotype characterized by biological interaction rather than simple coexistence [1].

The burden associated with COMISA exceeds that of either disorder alone. Compared with isolated insomnia or OSA, patients with COMISA demonstrate greater sleep fragmentation, reduced sleep efficiency, lighter sleep-stage predominance, and higher rates of psychiatric comorbidity [2, 3]. In neurological and psychiatric populations, the combined phenotype has been associated with poorer global functional status and greater overall impairment, suggesting that COMISA may act as a disease modifier rather than merely a comorbid diagnosis [4]. Longitudinal data further demonstrates steeper decline in work ability among individuals with COMISA compared with those with either disorder independently, highlighting broader societal implications [5]. Longitudinal population-based analyses further demonstrate steeper declines in work ability among individuals with COMISA compared with those with either disorder independently, reinforcing broader socioeconomic implications (6). COMISA is strongly associated with reduced work ability, with individuals at high risk for COMISA having 5.7 times higher odds of poor work ability compared to those without sleep disorders [5]. The impact was most pronounced in workers below 60 years, white-collar workers, and daytime workers.

Phenotypic heterogeneity further complicates clinical management. COMISA presentations vary according to objective sleep duration, psychiatric comorbidity, autonomic profile, and OSA endotype characteristics, and these differences influence treatment adherence and response. Baseline insomnia severity predicts poorer positive airway pressure adherence, while positive airway pressure (PAP) adherence influences insomnia trajectory, reflecting bidirectional interaction between respiratory stabilization and sleep continuity. Collectively, available evidence supports conceptualizing COMISA as a phenotype defined by mechanistic convergence between ventilatory instability and chronic hyperarousal, expressed through heterogeneous clinical presentations and associated with amplified cardiometabolic and functional burden. An integrated framework is therefore required to understand its epidemiology, mechanistic underpinnings, and clinical implications.

## Epidemiology and Clinical Burden

The epidemiology of COMISA is shaped by diagnostic methodology, sampling strategy, and phenotypic definition. Population-based studies show COMISA prevalence ranges from 1.7% to 11.4% depending on geographic region and diagnostic criteria used [6]. Community-based prevalence estimates are lower but remain clinically meaningful, with approximately 2–10% of adults meeting criteria for both chronic insomnia and moderate-to-severe OSA depending on diagnostic thresholds and age distribution [2, 7]. Variability across studies reflects differences in insomnia definitions (symptom-based versus DSM-5/ICSD-3 disorder), apnea–hypopnea index cutoffs, and testing modality, as home sleep apnea testing may underestimate disease severity in fragmented sleepers [8].

Age and sex influence COMISA expression. OSA prevalence increases with advancing age, whereas insomnia symptoms demonstrate a stronger female predominance even at comparable apnea severity, suggesting sex-related differences in hyperarousal vulnerability [2]. Other predictors of COMISA include sleep aid use, male sex, alcohol use, higher BMI, depression, and lower habitual sleep duration [9]. Comparatively, the overlap of insomnia with OSA in the pediatric population demonstrates developmental distinctions. In children referred for sleep evaluation, COMISA has been observed in approximately 13–27% of cases; [10] however, insomnia does not consistently correlate with respiratory event severity, and anxiety-related mechanisms appear to play a more prominent role than in adult cohorts. These findings suggest that COMISA is not a uniform entity across the lifespan.

Cardiometabolic risk represents one of the most concerning epidemiologic signals in COMISA. COMISA has emerged as a particularly potent contributor to new-onset hypertension and cardiovascular disease, with risks that exceed those observed in OSA alone [11, 12]. COMISA has been associated with a 47% increase in all-cause mortality and a 75% greater likelihood of prevalent cardiovascular disease relative to either disorder in isolation [13, 14], underscoring the prognostic significance of this combined phenotype. In hypertensive cohorts, the coexistence of insomnia and OSA has been independently linked with elevated predicted 10-year cardiovascular risk after multi-variable adjustment, whereas either condition alone does not consistently retain independent association [15]. Comparable findings have been observed in patients with type 2 diabetes, where COMISA remains significantly associated with high cardiovascular risk scores in fully adjusted models [16]. An additional concern is the reduced effectiveness of antihypertensive therapy in this population. Patients with COMISA often require more antihypertensive agents and have up to a threefold higher likelihood of developing resistant hypertension compared with those with OSA alone [17]. A consistent observation across epidemiologic studies is the dissociation between subjective symptom reporting and objective physiologic disturbances. Defining insomnia subtypes based on clinical characteristics, standardized questionnaires, or quantitative EEG features does not reliably identify individuals at highest risk of adverse outcomes [18, 19]. In contrast, phenotypes based on sleep duration have been found to characterize increased health risks more reliably. Accumulating evidence shows that two insomnia phenotypes — insomnia with PSG-measured short sleep duration (ISSD) and insomnia with PSG-measured normal sleep duration (INSD)—differ in their pathophysiologic mechanisms, natural course, cognitive-emotional characteristics, and associated adverse health outcomes [20]. Although the threshold commonly used to separate these phenotypes ranges between 4 and 6 h [21–23], insomnia of short sleep duration (ISSD) but not insomnia of normal sleep duration (INSD) demonstrates stronger associations with cardiometabolic and biomarker-defined risk, suggesting that reduced total sleep time represents a biologically distinct subgroup [24, 25]. These findings indicate that objective sleep metrics provide more meaningful stratification of disease burden than symptom severity alone.

## Clinical Presentation and Diagnostic Challenges

Clinical manifestations of COMISA are often difficult to categorize within traditional diagnostic boundaries. Individuals affected by this combined phenotype experience an array of daytime and nocturnal disturbances characteristic of both insomnia and OSA, and these manifestations frequently interact in a synergistic manner. The resulting clinical picture is one of heightened physiological strain, impaired daytime functioning, and significant deterioration in overall health and well-being [3, 26]. Many patients demonstrate sleep–wake misperception, frequently overestimating wakefulness and underestimating total sleep time, reflecting the cognitive and perceptual distortions commonly observed in chronic insomnia [27].

In general, individuals with COMISA often experience pervasive fatigue, diminished stamina, and impaired alertness [28, 29]. These symptoms rarely map cleanly onto a single disorder, as both insomnia and sleep-disordered breathing contribute to the overall presentation. In contrast to patients with OSA alone, who typically present with excessive daytime sleepiness, those with COMISA more frequently describe persistent tiredness, mental exhaustion, and cognitive inefficiency [12]. These symptoms likely reflect the combined effects of sleep fragmentation and an inability to achieve restorative deep sleep due to sustained hyperarousal [30, 31]. By comparison, nighttime sleep in COMISA is marked by pronounced instability. Patients commonly report difficulty initiating sleep, frequent nocturnal awakenings, and markedly reduced sleep efficiency. Further, COMISA patients experience more persistent sleep-maintenance difficulties and a higher frequency of early-morning awakenings [32, 33]. These disturbances are intensified by the chronic hyperarousal that defines insomnia, which lowers the arousal threshold and amplifies sympathetic responses to respiratory events during sleep [30, 34]. In neurologic and psychiatric populations, poor global functional status, including greater disability following stroke and more severe symptom burden in mood and seizure disorders has been reported in COMISA patients, suggesting that the combined phenotype may exacerbate underlying neurologic vulnerability [30]. Patients with COMISA exhibit deficits in attention, working memory, and processing speed, along with increased vulnerability to cognitive overload [35]. Emotional symptoms are similarly heightened; anxiety and depressive features occur more frequently and with greater severity than in individuals with OSA alone, contributing to reduced quality of life and diminished capacity to manage daily stressors [31]. Evidence from veteran populations demonstrates a strong association between insomnia severity, post-traumatic stress disorder, and fragmented sleep, with these factors contributing to increased overall symptom burden [36]. Both the INSD and ISSD phenotypes are associated with increased suicidal ideation, while the INSD phenotype is associated with an earlier age of onset of suicidality and a greater likelihood of hospitalization following suicide attempts [37].

The difficulty in early diagnosis is compounded by the limitations of commonly used screening tools. Instruments such as the Insomnia Severity Index and Epworth Sleepiness Scale were developed to assess individual conditions and therefore do not fully capture overlapping clinical presentations [38, 39]. As a result, patients with significant insomnia symptoms may not report excessive daytime sleepiness, leading to underrecognition of obstructive sleep apnea, while others with substantial sleep fragmentation may not meet conventional thresholds for insomnia despite meaningful impairment. The combined effects of hyperarousal and disrupted sleep architecture reduce the sensitivity of these tools in identifying coexisting diseases.

Objective testing introduces a different set of challenges. Polysomnography remains the standard for evaluating respiratory events and sleep structure, yet it does not reliably reflect the subjective experience of insomnia, particularly in individuals with conditioned arousal or sleep-state misperception [40]. Conversely, patients with severe perceived sleep disturbance may demonstrate relatively preserved objective sleep parameters, creating a disconnect that complicates interpretation. Although actigraphy and multi-night monitoring can provide insight into variability in sleep patterns, these approaches are not routinely integrated into diagnostic workflows [41]. The resulting gap between subjective and objective findings contributes to frequent misclassification and uncertainty in clinical decision-making.

## Pathophysiology and Mechanistic Convergence

The pathophysiology of comorbid insomnia and obstructive sleep apnea (COMISA) reflects the convergence of ventilatory control instability underlying OSA and sustained neurocognitive hyperarousal underpinning chronic insomnia within a self-reinforcing physiological framework. Rather than representing a simple coexistence of two independent disorders, COMISA embodies a bidirectional and self-reinforcing physiological state that amplifies sleep disruption, autonomic dysregulation, and cardiometabolic risk.

### Ventilatory Control Instability and Arousal-Driven Sleep Fragmentation

OSA arises from the interplay of several physiological traits, including elevated loop gain, impaired pharyngeal dilator muscle responsiveness, unfavorable upper-airway anatomy, and a low respiratory arousal threshold. These traits predispose individuals to recurrent upper-airway collapse, intermittent hypoxemia, and repetitive respiratory-related cortical arousals. In contrast, insomnia is characterized by persistent cortical and autonomic activation, with sustained engagement of wake-promoting neural networks and elevated sympathetic tone [1]. In COMISA, these arousals serve not only as markers of ventilatory instability but also as conditioning stimuli that reinforce insomnia-related wakefulness and maladaptive cognitive–behavioral responses to sleep. Apneas and hypopneas trigger repeated cortical arousals that fragment sleep and may condition wakefulness through reinforcement of maladaptive sleep–wake associations. Over time, this process can sustain insomnia symptoms even when respiratory events are adequately controlled. Patients with high loop gain may be particularly vulnerable to sleep-loss–induced ventilatory instability, whereas those with predominantly anatomical airway compromise may exhibit less modulation by hyperarousal traits [42]. Conversely, insomnia-related sleep restriction and fragmentation may alter ventilatory stability through changes in arousal threshold and control of breathing, thereby increasing susceptibility to obstructive events [43]. This bidirectional relationship helps explain why symptom burden and functional impairment in COMISA do not correlate directly with apnea–hypopnea index alone.

### Anatomic Dysregulation

Autonomic regulation represents a key interface through which these processes converge. Repeated cycles of hypoxia-reoxygenation, intrathoracic pressure fluctuations, and cortical arousals activate sympathetic pathways and the hypothalamic–pituitary–adrenal axis, promoting endothelial dysfunction, vascular stiffness, and insulin resistance [44]. Insomnia further contributes to autonomic imbalance through increased cortisol secretion and proinflammatory signaling [32]. Together, these mechanisms provide a biologically plausible explanation for the elevated prevalence of resistant hypertension, coronary artery disease, heart failure, stroke, and all-cause mortality observed in COMISA populations [14]. Autonomic data provides further physiologic support for this convergence model. Heart rate variability analyses show that individuals with COMISA exhibit heightened sympathetic predominance and delayed autonomic recovery following respiratory-related cortical arousals compared with AHI-matched patients with OSA alone [45, 46]. The persistence of sympathetic activation beyond the respiratory event suggests that arousal propagation in COMISA extends past transient event-linked disruption, reflecting sustained hyperarousal embedded within cardiovascular regulatory networks. This autonomic amplification offers a mechanistic bridge between sleep fragmentation and the elevated cardiometabolic risk observed epidemiologically [24, 47].

### Sleep Architecture and Neurostructural Disruption

In healthy adults, sleep architecture is characterized by consolidated NREM–REM cycles, with progressive deepening into slow-wave sleep (N3) early in the night and REM-rich periods later [48]. In COMISA, this architecture is eroded from two directions. OSA drives repetitive microarousals and sleep fragmentation, often reducing slow-wave sleep and sometimes REM duration, while increasing lighter N1 and N2 stages. Insomnia, in turn, adds difficulty initiating sleep and reinitiating it after arousals, amplifying wakefulness between and after respiratory events [49]. The result is a night marked by frequent transitions between N1, brief N2, and wake, with unstable or truncated N3 and REM periods and a high microarousal index. In COMISA, quantitative electroencephalographic analyses reveal persistent high-frequency (beta) activity and reduced slow-wave power during non–rapid eye movement sleep [50], consistent with insomnia-related cortical hyperactivation super-imposed on OSA-related instability. This pattern indicates incomplete deactivation of wake-promoting circuits alongside repeated respiratory perturbation. The coexistence of elevated sleep-pressure markers and hyperarousal signatures suggests that COMISA is defined by instability across both arousal and ventilatory domains rather than dominance of one mechanism.

Emerging neuroimaging data further suggests structural vulnerability. Gray matter alterations within temporal and limbic regions have been reported with increasing COMISA severity, implicating networks involved in memory consolidation and emotional regulation [51]. Given that both intermittent hypoxia and sustained sympathetic activation promote oxidative stress and neuroinflammation, the convergence phenotype may predispose to accelerated neural injury relative to either condition alone.

## Treatment Strategies and Outcomes

The diagnostic complexity of COMISA has direct implications for management, as effective treatment requires simultaneous consideration of both insomnia and sleep-disordered breathing rather than prioritization of a single domain. Approaches that target only respiratory abnormalities or subjective sleep complaints frequently result in incomplete clinical improvement, supporting the need for integrated and individualized treatment strategies [2, 20].

## CPAP Adherence and the Role of Comorbid Insomnia

Despite its established efficacy, CPAP adherence is substantially compromised in patients with comorbid insomnia. Multiple converging lines of evidence illuminate both the magnitude and the mechanisms of this problem. Recent evidence suggests a bidirectional relationship in which patients adherent to CPAP (≥ 4 h per night) experienced significantly larger reductions in insomnia severity compared with non-adherent patients, while higher baseline insomnia severity independently predicted non-adherence [52]. Others identified sleep-onset insomnia, sleep-maintenance difficulties, anxiety-related hyperarousal, and claustrophobia as key drivers of CPAP rejection and reduced long-term use [53]. These findings are corroborated by Ragnoli and colleagues [2], concluding that untreated insomnia—particularly hyperarousal and sleep-onset difficulties—is among the strongest predictors of poor PAP adherence and overall treatment response in COMISA. At the same time, practical factors such as mask discomfort, air leakage, and device noise can reduce PAP tolerance in patients with comorbid insomnia, limiting the extent to which PAP therapy alone can alleviate their symptoms.

Interestingly, the problem of poor adherence to treatment is not device-specific: Kaffenberger and colleagues [54] reported that patients with COMISA using upper airway stimulation utilized the device for significantly fewer hours per night than OSA-only controls and paused therapy more frequently, with insomnia remaining an independent predictor of both outcomes after adjustment for sex.

### Cognitive Behavioral Therapy for Insomnia (CBT-I)

CBT-I is the recommended first-line treatment for chronic insomnia disorder [55]and has demonstrated meaningful efficacy within the COMISA population [56]. CBT-I has been shown also in randomized trials to modestly reduce apnea severity, likely through improved sleep consolidation and increased slow-wave sleep [2, 56, 57]though it does not modify intrinsic ventilatory endotypes such as loop gain. The effect of CBT-I on improving PAP adherence may be most pronounced in patients with at least moderate OSA, and when the CBT-I intervention is administered by trained clinicians, rather than through a static self-guided reading intervention [56]. A recent meta-analysis of 21 studies demonstrated a large pooled effect size for insomnia improvement, with even greater effects in untreated OSA compared with treated OSA, supporting CBT-I as both a standalone and adjunctive therapy [56]. These findings reinforce that insomnia-targeted therapy can substantially reduce hyperarousal even when respiratory instability persists. However, the remission rate with CBT-I in COMISA is generally lower than in insomnia-only populations, and there is sizable variability in individual treatment responses. The mechanisms behind this reduced efficacy likely reflect the biological complexity of COMISA—specifically, the interplay between OSA-related arousals, hyperarousal states, and the behavioral components that CBT-I primarily targets.

Emerging literature suggests that one-size-fits-all CBT-I is insufficient for COMISA. Pre-treatment phenotyping — specifically measuring objective TST, screening for PTSD, assessing baseline sleepiness, and considering cultural and demographic factors — should inform whether CBT-I is appropriate, whether pharmacological augmentation is warranted, or whether a more comprehensive CBT-I model is needed. Our group has recently identified that total sleep time (TST) is an important predictor of response to behavioral treatment. Although the study population comprised veterans exclusively, a TST threshold of ≥ 4.1 h was found to be the optimal cut-point for predicting behavioral treatment response [22] (Table1). Other studies reached similar conclusions [23]. The clinical significance of the TST threshold is mechanistically grounded. The core of CBT-I relies on consolidating fragmented sleep through controlled sleep restriction; imposing further restrictions on patients who are already sleeping fewer than 4.1 h per night may paradoxically limit the treatment’s effectiveness by depriving them of the restorative sleep necessary to benefit from behavioral interventions. Moreover, insomnia of short sleep duration (ISS) is associated with elevated physiological hyperarousal—characterized by increased cortisol, heart rate, and sympathetic nervous system activity—a biological state that CBT, which primarily targets cognitive and behavioral factors, may not adequately address [11]. These findings introduce a novel conceptual framework suggesting that insomnia phenotyping prior to treatment selection could meaningfully improve outcomes and resource allocation in COMISA.

### Neurocognitive Considerations During CBT-I Initiation

A clinically important safety signal has emerged from comparative treatment research. CBT-I-containing pathways may be associated with transient deterioration in vigilance and executive functioning during the acute treatment phase. Turner and colleagues [58] observed poorer objective neurocognitive performance in CBT-I groups relative to PAP alone, raising concerns about short-term functional impairment. This consideration is particularly relevant for COMISA patients in safety-sensitive occupations—commercial transportation, aviation, heavy machinery operation—where even brief lapses in vigilance carry significant risk. Because obstructive sleep apnea is frequently accompanied by marked daytime sleepiness, there is understandable concern that implementing sleep restriction therapy in individuals with COMISA could further heighten sleepiness to a degree that becomes unsafe, particularly given the well-documented association between OSA-related sleepiness and motor vehicle accident. Given the public health concerns, clinicians should incorporate functional context and occupational demands into treatment sequencing decisions and may need to implement compensatory safety measures during the early stages of behavioral insomnia treatment.

### Pharmacological Approaches

The role of sedative-hypnotic agents in COMISA management requires careful individualization. Benzodiazepines and Z-drugs carry specific concerns regarding respiratory drive suppression and psychomotor impairment, making them suboptimal first-line agents in COMISA—particularly in patients with severe OSA. Longer-term use of gamma-aminobutyric acid (GABA) receptor agonists also raises concerns regarding tolerance, dependence, sleepwalking, and falls, generally limiting their recommended use to short-term courses (≤ 4 weeks) [59]. Dual orexin receptor antagonists (DORA) approved for the treatment of insomnia disorder in adults, have emerged as a pharmacological option of particular interest in COMISA given their distinct mechanism of action [60]. Unlike GABA-agonist hypnotics that broadly suppress neurological activity, DORAs selectively block the binding of orexin A and B to their receptors, attenuating overactive wake-promoting signaling while preserving more physiologically normal sleep architecture [61]. This mechanistic profile may be advantageous in patients with OSA, as it avoids the respiratory depression concerns associated with benzodiazepines and Z-drugs [62].

Lettieri and colleagues [60] conducted a post hoc subgroup analysis of a Phase 3 randomized, double-blind, placebo-controlled trial evaluating daridorexant 25 mg and 50 mg in adults with comorbid insomnia disorder and untreated mild OSA. This represented the first formal evaluation of daridorexant in the COMISA population. The study subgroup was older (mean age 63.8 years), predominantly male (39.9%), and had moderate-to-severe insomnia at baseline (mean ISI 18.5). Notably, baseline wake after sleep onset (WASO) was prolonged compared with the overall Phase 3 population (mean 114.2 vs. 98.6 min), consistent with the known difficulty of maintaining sleep in the setting of comorbid OSA. Daridorexant 50 mg significantly improved WASO from baseline at both Months 1 and 3, and self-reported total sleep time improved significantly at both timepoints. The average treatment effect size for all efficacy parameters was numerically greater with daridorexant 50 mg than 25 mg, though the 25 mg dose did not achieve statistical significance at either timepoint for WASO or latency to persistent sleep.

Of clinical importance, daridorexant 50 mg also showed a trend toward improvement in daytime functioning as assessed by the Insomnia Daytime Symptoms and Impacts Questionnaire total score, though between-group differences from placebo were not statistically significant. Critically, no worsening of next-morning sleepiness was observed with either dose; in fact, a trend toward reduced morning sleepiness was seen across all treatment arms, with numerically greater improvement with daridorexant 50 mg. ESS scores remained within the normal range throughout the study, and no dose dependency in adverse events—including daytime somnolence—was observed. The safety profile of participants with mild OSA was similar to that of the overall Phase 3 study population, and no participants discontinued treatment due to adverse events.

Melatonin has emerging evidence supporting its use in individuals with comorbid insomnia and OSA, particularly those who remain symptomatic despite PAP therapy. A recent randomized, placebo-controlled trial in PAP-adherent COMISA patients demonstrated that melatonin 10 mg improved insomnia severity, sleep quality, daytime functioning, and PAP adherence without worsening respiratory parameters [63]. These findings align with prior work showing altered melatonin secretion patterns in OSA and suggest that melatonin may help reduce hyperarousal and circadian dysregulation in this population.

### Combining Pharmacotherapy with Cognitive Behavioral Therapy

An alternative pharmacological strategy—augmenting behavioral therapy with a short course of hypnotics to accelerate initial response—has been evaluated in the highly treatment-resistant COMISA population of veterans with post-traumatic stress disorder (PTSD). El-Solh and colleagues [64] conducted a pilot open-label randomized controlled trial comparing brief behavioral therapy for insomnia (BBTI) alone versus BBTI augmented with a two-week course of eszopiclone (2 mg nightly) in 53 PTSD veterans with COMISA, all of whom were non-adherent to CPAP at baseline (defined as < 4 h per night). Both treatment arms achieved significant reductions in sleep quality impairment (Pittsburgh Sleep Quality Index), insomnia severity, daytime sleepiness, depression, and PTSD burden from baseline to 24 weeks with no statistically significant group-by-time interaction effects. This indicates that BBTI alone and BBTI plus eszopiclone were comparably effective over the full 24-week follow-up period. However, the combination arm demonstrated a significantly higher insomnia remission rate at the 6-week follow-up (31% vs. 7%), suggesting that eszopiclone conferred a meaningful early benefit in accelerating remission—a finding with potential clinical relevance for patients requiring more rapid functional recovery. There was also a notable impact on PAP utilization. During the initial 6-week treatment phase, patients in the BBTI plus eszopiclone arm showed a significant increase in PAP use from baseline, whereas no significant change was observed in the BBTI-only group. Patients receiving combination therapy used PAP on average 75.8 min more per night than BBTI-only patients at week 6. However, this PAP adherence advantage was not sustained at the 24-week follow-up, and by that endpoint, both groups showed significant increases in PAP utilization from baseline. Although the study was conducted exclusively in male veterans with PTSD, the observed benefits of short-term hypnotic augmentation—particularly the acceleration of early insomnia remission and transient improvement in PAP adherence—target core behavioral and physiological mechanisms common to COMISA across populations. As such, while replication in more diverse cohorts is needed, these findings may cautiously inform treatment strategies for non-veteran patients with severe, treatment-resistant COMISA who require rapid symptom relief to facilitate engagement with behavioral and PAP-based interventions.

### Sequential Treatment Approaches

The weight of available evidence supports integrated management of COMISA—treating both insomnia and OSA concurrently or in close sequence—rather than defaulting to PAP therapy alone. The bidirectional interaction between the two conditions means that treating only one component frequently results in suboptimal outcomes for both. Ragnoli et al. [2]concluded that integrated management yields superior improvements in adherence, sleep quality, and daytime functioning compared with single-modality approaches. The El-Solh study [64] provides additional mechanistic support: BBTI responders demonstrated substantial gains in PAP utilization that non-responders did not, confirming that successful insomnia management is not merely a parallel goal but a prerequisite for optimal PAP adherence in many patients.

In practice, the optimal treatment sequence will depend on patient-specific factors. For patients with predominant sleep-onset insomnia and mild to moderate OSA, initiating CBT-I (or BBTI) prior to or concurrently with PAP may improve PAP acceptance by reducing the pre-sleep hyperarousal and prolonged sleep onset latency that heightens sensitivity to the pressurized CPAP interface. For patients with severe symptomatic sleep apnea and in whom neurocognitive impairment or occupational safety is a primary concern, initiating PAP first and introducing behavioral insomnia treatment after airway stabilization may be preferable, given the transient cognitive risks associated with CBT-I initiation. For patients with very short sleep duration (TST < 4.1 h), standard CBT-I protocols may be insufficient, and clinicians should consider augmented approaches—including short-course pharmacotherapy or modified sleep restriction protocols—tailored to the ISS phenotype.

## Conclusion

COMISA should no longer be conceptualized as the simple coexistence of two prevalent sleep disorders. Converging evidence from physiologic, autonomic, neurocognitive, and cardiometabolic domains supports a model of interactive amplification in which ventilatory instability and chronic hyperarousal reinforce one another through shared neural and autonomic pathways. This bidirectional coupling offers a coherent explanation for the disproportionate symptom burden, impaired treatment adherence, and heightened cardiovascular vulnerability observed in affected individuals. Recognition of this diversity reframes inconsistent treatment outcomes not as therapeutic failure, but as evidence of underlying biological variability. A phenotype-informed approach that integrates endotypic traits, objective sleep metrics, and autonomic markers may therefore represent the next logical evolution in COMISA management.

The field now stands at an inflection point. Moving beyond symptom-based classification toward biologically anchored stratification will require longitudinal outcome studies, endotype-stratified interventional trials, and incorporation of digital and biomarker-informed phenotyping. By embracing this convergence framework, future research can determine whether COMISA represents a distinct pathophysiologic entity or a spectrum of interacting traits requiring precision-targeted care.

## Figures and Tables

**Table 1:** Clinical characteristics of COMISA patients based on total sleep time

Characteristic	Better Responders	Poorer Responders
Total Sleep Time	≥ 4.1 h	< 4.1 h
Race	White/Caucasian	Non-white
Insomnia Duration	Shorter (trend)	Longer (trend)
Daytime Sleepiness	Lower baseline	Higher baseline
OSA Severity (AHI)	Not predictive	Not predictive

## Data Availability

No datasets were generated or analysed during the current study.
